# Using structural and functional brain imaging to uncover how the brain adapts to blindness

**Published:** 2015-08-13

**Authors:** Gabriella V. Hirsch, Corinna M. Bauer, Lotfi B. Merabet

**Affiliations:** The Laboratory for Visual Neuroplasticity, Department of Ophthalmology, Massachusetts Eye and Ear Infirmary, Harvard Medical School. Boston, MA, USA

**Keywords:** Plasticity, blindness, neuroimaging, MRI, crossmodal processing, ocular blindness, cortical visual impairment

## Abstract

Advances in neuroimaging technology have been instrumental in uncovering the dramatic neurological changes that result from blindness, as well as revealing the inner workings of the human brain. Specifically, modern imaging techniques enable us to examine how the brain adapts and “re-wires” itself as a result of changes in behavior, the environment, injury, or disease; a process referred to as neuroplasticity. Following an overview of commonly employed neuroimaging techniques, we discuss structural and functional neuroplastic brain changes associated with profound visual deprivation. In particular, we highlight how associated structural changes often occur within areas that process intact senses (such as hearing, touch, and smell) while functional changes tend to implicate areas of the brain normally ascribed to the processing of visual information. Evidence will primarily focus on profound blindness due to ocular cause, but related work in cerebral/cortical visual impairment (CVI) will also be discussed. The potential importance of these findings within the context of education and rehabilitation is proposed.

## Introduction

We are living in an extraordinary time in the history of neuroscience. Just over a century ago, our knowledge of the brain and its inner workings were largely limited to observations that could be made from examining post-mortem specimens. Today, our understanding comes from the ability to characterize the structure and function of the living brain with great detail, as well as in a safe and noninvasive manner. In this effort, the development and use of modern day brain imaging techniques have been transformative towards our understanding.

The brain is a highly organized organ; an amalgamation of many specialized regions, each associated with its own function including vision, hearing, touch, memory, speech, and emotion. Humans are highly visual creatures, and thus it is perhaps not surprising that a large proportion of the brain is responsible for the processing of visual information. In fact, cortical areas implicated with visual processing comprise roughly 30 to 40% of the brain’s cortical surface [1,2]. These areas are largely localized within the occipital cortex with higher order visual processing areas extending into the parietal, temporal, and frontal cortices. Within the visual brain, distinct regions can be further subdivided, each responsible for analyzing specific aspects of a visual scene such as form, color, and motion, as well as high level representations including faces and shapes [2].

Given the importance of visual information in characterizing the world around us, initial notions purported that the early onset of profound visual deprivation would likely have dramatic and detrimental effects on an individual’s learning and cognitive development [3]. However, considerable scientific evidence demonstrates that the brain is highly malleable throughout life, and can adapt its structural and functional organization in response to experience, development, the environment, and damage [4–6]. This adaptive ability of the brain is termed *neuroplasticity*. In the context of blindness, there is considerable interest geared to understanding how neuroplastic changes within the brain are linked to observed compensatory behaviors of the non-visual senses (e.g., hearing, touch, and smell) as well as cognitive skills (e.g., language, memory, and musical ability) [6–9]. While the exact nature of these adaptive behaviors is still the subject of intense investigation, key questions emerge in light of our understanding of how the brain is organized. For example, how do different parts of the brain (each responsible for their own type of information processing) change within the context of blindness? What is the fate of brain regions dedicated to processing visual information in a person who is born profoundly blind? Are these brain areas rendered silent, or do they serve other purposes? If they do take on other functions, how is this related to compensatory behaviors observed in the blind? Answering these questions has important implications not only in terms of our understanding of how individuals adjust to living without sight, but also with regards to the potential of the brain to develop and adapt to sensory deprivation. In this direction, brain imaging techniques have provided highly valuable insight.

## Review

### Imaging the structure and function of the brain: concepts and limitations

Conceptually, neuroimaging techniques can be classified into two approaches; structural and functional. Structural imaging refers to approaches that are specialized for the visualization and analysis of anatomical properties of the brain. Structural approaches are particularly useful for detecting brain damage and abnormalities. Furthermore, analyses can be performed to quantify geometric structural properties such as the size and volume of a given structure [10] or the thickness of a cortical area (e.g., grey matter) [11]. In contrast, functional imaging is used to identify brain areas and underlying brain processes that are associated with performing a particular cognitive or behavioral task. Depending on the type of signal being analyzed, inferences between the location of brain activity and brain function can then be determined [12–14].

While numerous neuroimaging techniques exist, it is important to realize that each possesses its own strengths and weaknesses (see Table 1 for summary). In this context, the concept of resolution needs to be highlighted. *Spatial resolution* refers to the ability of an imaging technique to distinguish between two points in space (or structures within the brain) that are in close proximity. The higher the spatial resolution, the finer the detail that can be provided. In contrast, *temporal resolution* refers to the ability to distinguish between two events occurring over a given time period. The higher the temporal resolution of an imaging technique, the better it can discriminate between two events occurring in close sequence. Typically, there is a trade-off between the two types of resolution in that an imaging technique with excellent resolution in one domain will do so at the expense of the other (i.e., high spatial resolution typically comes at the expense of low temporal resolution and vice versa). For this reason, comprehensive brain imaging studies will often employ a combination of techniques to leverage their respective strengths and provide complementary information [15].

Here, we provide a brief overview of the primary neuroimaging techniques used in clinical and/or research settings while highlighting their comparative strengths and weaknesses. We then show how these techniques have helped elucidate neuroplastic changes occurring in the brain of blind individuals and how they relate to non-visual compensatory behaviors. Special emphasis will be given to results from magnetic resonance imaging (MRI)-based modalities, as these have become the methods of choice in clinical research given their ability to provide highly detailed information for both structural and functional imaging studies.

### Common approaches to investigating brain structure and function

**Figure 1:**
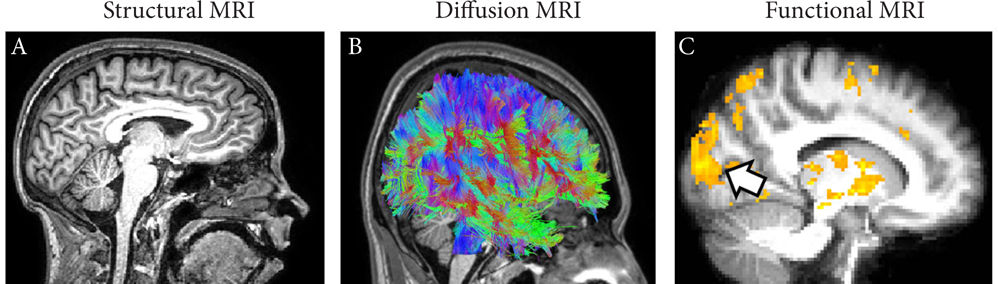
MRI based imaging techniques (data presented was obtained by the authors and is for illustrative purposes) (**A**) Structural MRI reveals the gross anatomical structure of the brain with high detail (shown in sagittal view). (**B**) Diffusion MRI reveals the overall layout of white matter connections and pathways within the brain (referred to as tractography). The color scheme corresponds to the orientation of the white matter fibers (green represents fibers extending between the front and back of the brain, red is for fibers running from left to right, and blue is for fibers along the axis from the top to the bottom of the brain). (**C**) Functional MRI reveals areas of the brain that are active when and individual is asked to perform a particular task. In this case, an individual who is congenitally blind was asked to identify a tactile pattern through touch. Note areas of activation identified throughout the brain (yellow) including within the occipital cortex which is normally associated with visual information processing (identified by the arrow).

Computed Tomography (CT) is a widely accessible structural imaging technique that uses highly focused X-ray beams to take multiple cross-sectional images of the brain [16,17]. CT scans are well suited to image the skull and bone as well as blood, and can generate images with good spatial detail and within a short period of time. However, CT is relatively poor in its ability to discriminate between different tissue types within the brain compared to MRI (see below) and involves exposing the patient to ionizing radiation (similar to that of a chest X-Ray procedure). Magnetic Resonance Imaging (MRI), on the other hand, provides highly detailed images of the brain. The superior spatial resolution of MRI comes from using strong magnetic gradients [18,19]. Tissues within grey and white matter (as well other parts of the brain) respond to these magnetic gradients differently, allowing for the differentiation of tissue types and structures (Figure 1A). Diffusion tensor imaging (DTI) is a variant of structural MRI that is able to provide detailed reconstructions of white matter connections *in vivo*, revealing how the brain is “wired”. DTI tracks the constrained movement of water molecules within the brain; the axis of this movement reflects the orientation of underlying white matter tracts [20]. Using sophisticated algorithms, this information can then be used to reconstruct the fine architecture of neural pathways along three dimensions, referred to as *tractography* (Figure 1B) [21].

Turning our attention to functional neuroimaging techniques, functional magnetic resonance imaging (fMRI), is another variant of MRI that tracks physiological changes in the brain (i.e., changes in blood flow and oxygen levels) associated with performing a cognitive or behavioral task [22]. That is, the “harder” a certain area of the brain works while performing a given task, the more metabolic demand that area has. This is referred to as the blood oxygen level dependent (BOLD) response [23], and regional changes in this signal can be detected by the scanner (Figure 1C). It should be noted that fMRI (unlike EEG, see below) is an *indirect* measure of brain activity based on the aforementioned physiological changes occurring within the brain [24]. During fMRI, the subject must lie perfectly still as head motion can compromise image quality. Furthermore, because of the strong magnetic fields used, individuals with non-removable ferromagnetic metal in the body (e.g., pacemakers) cannot be scanned using MRI [25]. Additional safety precautions must be observed including wearing ear protection due to the loud noises generated by the scanner. The electroencephalogram (EEG), directly records the brain’s underlying electrical activity using very sensitive electrodes placed on the surface of the scalp. Signals associated with localized neuronal activity are amplified and averaged so as to characterize the brain’s overall electrical activity [26]. When recordings are made from the occipital cortex in response to a visual stimulus, this is referred to as a visual evoked potential (VEP; more generally termed as an “event-related potential” or ERP) [27]. A related technique to EEG is magnetic electroencephalography (MEG) which measures minute electro-magnetic currents from the brain [28]. Its higher signal sensitivity affords it comparatively superior spatial and temporal resolution than EEG, and MEG has often been used in cortical mapping studies alongside more typical structural based imaging techniques ([29] later in the text). Positron emission tomography (PET) is another form of functional imaging which is similar to fMRI in that it is an indirect measure of neural activity in response to a task being performed. PET utilizes a radioactive tracer (typically a glucose analogue) to detect the amount of energy consumed by the brain [13]. Highly active brain regions have greater metabolic demand, resulting in an augmented uptake in glucose tracer and regional increases in the signal detected. The inverse is true for brain regions with diminished neural activity due to disease [30]. Compared to fMRI, PET imaging is very costly and has the prominent drawback of requiring the use of a radioactive tracer, inherently limiting the number of times a person can be scanned [17].

### Structural brain changes occurring as a result of blindness

Upon direct visual inspection, the brains of blind individuals do not appear to show gross anatomical differences compared to individuals that are normally sighted (albeit in the case where there is clear structural damage to the eye itself). However, taking advantage of the finer spatial resolution afforded by MRI, marked structural differences are indeed evident, particularly in brain regions involved in processing non-visual senses.

Among the first areas of the brain to be explored were regions associated with hearing and language. A number of studies have reported that the blind possess heightened auditory processing abilities such as enhanced sound localization [9,31–33] and even increased incidence of perfect pitch within the blind population [34]. In a study by Elbert and colleagues, regions of the brain responsible for the processing of auditory information showed marked structural changes within the context of blindness; specifically, there was an enlargement of the core areas of the auditory cortex [35]. These authors proposed that these changes were related to the greater reliance on auditory information in blind individuals. Along the same lines, Hamilton and colleagues found that the planum temporale (a cortical region located behind the primary auditory cortex and important for language and music processing) was larger in blind musicians with absolute pitch compared to sighted musicians with the same skill [34].

A similar story appears with regards to the sense of smell, whereby an increased volume of the olfactory bulb (the principle brain structure implicated in human olfaction) has been observed. As in the case for auditory processing areas, the size of the olfactory bulb was found to be larger in the early blind as compared to age and gender-matched sighted controls. In fact, performance on an odor discrimination task correlated with olfactory bulb size (i.e., the better an individual was at identifying smells, the larger the olfactory bulb) [36,37].

Touch is also a sense for which blind individuals rely on heavily, particularly within the context of braille reading. In an intriguing study, Sterr et al., (1998) used MEG (though technically, a functional neuroimaging technique) to map out the cortical representation of the individual fingers in brain. This group showed that in blind proficient braille readers who used multiple fingers to read text, the cortical representation of the fingers appeared to overlap or “fuse” as opposed to being individually segregated as in the case of normally sighted subjects. These combined structural-functional changes observed in the blind were interpreted to reflect enhanced tactile and holistic processing abilities related to more efficient tactile reading skills [29].

Finally, memory processing and way-finding abilities have also been shown to be associated structural changes at the level of the brain. The hippocampus is a critical structure for memory function and the same time also plays a key role in spatial navigation and way finding abilities [38,39]. In one study, Fortin and colleagues (2008) found that blind individuals showed superior abilities (compared to sighted controls) in learning new maze paths and enhanced recognition of small-scale tactile maps representing the spatial environments explored. Furthermore, in the same study, the authors also found that the hippocampus was larger in the blind compared to sighted control subjects [40].

That is the significance of these associated structural changes, and in particular, the larger size of structures associated with non-visual skills? These changes can be explained within the context of use-dependent plasticity. Indeed, as noted previously, a defining feature of the brain is its ability to change and adapt its structure in response to experience. In the case of visual impairment, it is suggested that these changes occur based on an increased use and a greater reliance on nonvisual senses (such as hearing, smell and touch) and skills (e.g., memory). As a result, this structural plasticity appears to be associated with non-visual sensory skills within the context of behavioral compensations [9,41].

Lastly, we turn our attention to areas of the brain normally associated with the processing of visual information. Specifically, there have also been a number of studies investigating structural differences in the sensory cortices (including visual, auditory and somatosensory cortices) of blind individuals. In contrast to brain areas implicated with the processing of non-visual information, structural changes observed in visual cortical areas have been less clear. In fact, current literature appears divided in that some studies report increases in grey matter structures of primary visual cortical areas, while others have reported decreases of the same areas. Similarly, identification of white matter changes have been observed whereby decreases in grey matter can be concomitant with increases in the white matter of those same areas [42–48]. Diffusion-based MRI techniques such as DTI have been used to investigate the underlying white matter networks that connect various regions of the brain. In individuals who are profoundly blind since birth, diffusion-based tractography has revealed a number of changes to white matter structures in early blind individuals implying a neural “rewiring” [49]. Some findings report an overall atrophy (i.e., a reduction in structural size) of the white matter fibers carrying information from the eyes, starting from the optic nerves to the optic radiations, and eventually terminating in the occipital cortex [48]. Further, Shu et al., (2009) found that parts of the brain responsible for motor and somatosensory functions appeared to have increased connections with other non-somatosensory brain regions in blind individuals. The authors concluded these differences may be the result of use-dependent changes supporting the processing of non-visual information in a more efficient manner [50].

Within the context of use dependency plasticity, reported changes in grey matter morphology could reflect typical developmental as well as compensatory plasticity which are likely influenced by the lack of visual input during early developmental periods [47]. Indeed, the complex interplay of genes and experiences likely contribute to the development of these reported discrepancies, the clarification of which are further hindered by differences in study methodology and/or sample population (e.g., age, gender, onset, and degree of visual function). However, a more careful analysis of brain structure and connectivity are likely to provide clues as this work moves forward.

In summary, the changes in brain structure that occur with blindness appear to support the concept of use-dependent plasticity. As blind individuals have a greater reliance on information garnered from non-visual senses, there appears to be a concurrent increase in the size of brain structures supporting non-visual functions such as hearing, touch and smell. In addition, the overall connectivity of the brain appears to be altered. This may perhaps support greater efficiency of information processing between parts of the brain responsible for non-visual senses and tasks associated with enhanced compensatory behaviors. Therefore, while blindness also appears to be concomitant with structural changes in cortical areas normally implicated in visual processing; the implications of these developmental modifications are less clear. To reveal the contribution of visual areas in relation to compensatory behaviors in blind individuals, we now turn to results from functional neuroimaging studies.

### Functional changes occurring as a result of blindness

Structural imaging has revealed changes in brain regions responsible for processing non-visual information. However the associated changes in brain areas normally responsible for visual processing remain less evident. Specifically, the question still remains as to what becomes the fate of brain areas normally related to the processing of visual information in the setting of profound blindness.

One of the first functional neuroimaging experiments examining the activity of the occipital cortex in the blind was conducted in 1990 by Veraart and colleagues using PET imaging. In this study, PET revealed that the occipital cortex was more metabolically active in individuals who were born blind compared to individuals who were normally sighted (keeping their eyes closed). This result was important in providing the first clue that occipital cortical areas were indeed somehow active following profound visual deprivation [51]. While intriguing, this finding did not speak as to whether this increased metabolic activity was associated with any particular task or behavior. To answer this question, Sadato et al., (1998) used PET to study occipital cortex activation while blind individuals read braille text. In this study, the task of braille reading was found to be associated with strong activation within occipital cortical areas, demonstrating for the first time that in the case of blindness, areas of the brain normally associated with the processing of visual information are recruited to process information related to nonvisual sensory modalities (in this case, touch) [52]. These initial findings showing occipital activation in response to reading braille have since been replicated by many groups [6,53,54]. Specifically, more recent studies have leveraged the greater spatial resolution of fMRI in order to investigate whether the occipital cortex is active in other types of nonvisual tasks. Beyond the task of braille reading, a number of studies have reported that the blind outperformed sighted controls on a wide range of texture discrimination tasks, including tactile acuity [55], shape discrimination [56], groove space discrimination [57], and tactile symmetry detection [58]. The significance of this activation may be related to role that these areas play in the processing of elemental visual features such as points, lines, and geometrical shapes. Thus, in the context of blindness, it appears that early visual areas are also implicated in the processing of simple lines and patterns when they are explored through touch. This observation is also evident in the higher order visual processing areas. For example, the lateral occipital complex (LOC), which is implicated in visual shape perception, is also active when blind individuals explore complex tactile shapes [59]. The middle temporal area (area MT or V5) is specialized for motion processing and is also active when blind individuals perceive motion from a moving tactile stimulus [60]. Finally, areas implicated with the processing of facial features are also active when the blind explore tactile face masks [61]. The significance of these results is that the cortical specialization of feature processing appears to be retained across modalities, even in the setting of blindness.

As mentioned previously, superior sound localization and identification abilities have been reported in blind individuals compared to sighted counterparts [9]. Using the superior temporal resolution of EEG [62] and spatial resolution of fMRI [63], there is corroborative evidence that the occipital cortex is also active in blind individuals while they perform sound localization and identification tasks.

Investigations of brain activity related to chemical senses in the blind have also been carried out, suggesting that the processing of smell implicates the recruitment of occipital cortical areas. In line with the structural changes observed in olfaction processing areas of the brain, behavioral enhancements have been reported as well. For example, one study found that blind individuals were better at identifying and discriminating smells compared to sighted controls [64]. However, it is worth noting that other groups have proposed that the blind have a lower threshold of odor perception without the increased ability to discriminate between different smells [65,66]. Despite this discrepancy, fMRI results have shown that when blind participants identify various odors (regardless of task), associated activation within occipital cortical areas is evident [67].

Finally, there have been a number of reports describing superior memory skills in blind individuals (e.g., [68,69]. Certain types of memory functions, such as serial memory (i.e., the ability to recall the order in which items are encountered) are especially important in everyday functions. Interestingly, while memory recall typically does not involve direct input from the senses (such as in the case of touch, hearing, or smell) neuroimaging studies using fMRI have shown that in the blind, there is increased activation within primary visual cortical areas during verbal-memory tasks. Furthermore, and of further interest, it was noted that the magnitude of this activation was correlated with an individual’s recall performance on the memory task [70].

The recruitment of occipital visual areas for the processing of nonvisual sensory information is referred to as *cross-modal plasticity*. Crossmodal plasticity can be defined as a process in which brain areas usually devoted to processing one type of sensory input (e.g., visual information within the occipital cortex) are recruited to process information relating to another sensory modality (e.g., tactile or auditory information) [71]. Current notions suggest that in parallel to the observed structural and connectivity changes outlined earlier, functional changes (i.e., the crossmodal recruitment of visual cortical areas for the processing of non-visual sensory information) may also be intimately related to compensatory, and in some cases even superior, behavioral skills observed in blind individuals [6,41].

The ability of the brain to reorganize itself within the setting of visual deprivation is indeed a remarkable example of developmental neuroplasticity. As described above, a number of neuroimaging studies have demonstrated that the occipital visual cortex is active while the blind perform a variety of non-visual tasks. This has led to the prevailing view that compensatory (and even superior) behaviors observed in the blind are the result of crossmodal sensory processing within the visual cortex and changes in the overall functional organization of the brain.

Causal evidence for the functional relevance of the occipital cortex in non-visual sensory processing has come from both clinical as well as experimental accounts. For example, a report by Hamilton and colleagues [72] describes the case of a congenitally blind and highly proficient Braille reader who became alexic for Braille following bilateral damage to her occipital cortex following a stroke. Experimental evidence has also arisen from the use of transcranial magnetic stimulation (TMS) as a means to non-invasively disrupt the activity of the visual cortex and observe its impact on behavioral performance [73]. For instance, transient and reversible disruption of occipital cortical areas of the blind disrupts their performance on a variety of tasks including Braille reading [53] and auditory localization [74]. Intriguingly, other cognitive tasks such as verbal processing have also been shown to be disrupted following reversible disruption of occipital cortical areas [75].

In line with the view that the occipital cortex supports the crossmodal processing of non-sensory information, it would seem reasonable to expect concomitant patterns of brain reorganization reflecting an enhancement in connectivity between occipital cortex and areas responsible for auditory and somatosensory processing. Indeed, analyses of predetermined (i.e., based on prior assumptions) regions of interest (ROIs) support this view of enhanced connectivity between occipital cortex areas and regions responsible for auditory [76] as well as tactile processing [77,78].

More recently, whole-brain resting state functional connectivity MRI (or rsfcMRI) analysis has received increasing interest for its ability to characterize functional connections between cortical areas [79] and give a more complete view of the effects of visual deprivation on the overall organization of the brain. While it is important to note that rsfcMRI is not a direct measure of anatomical connectivity, inferences regarding the strength of connections between large-scale systems can be made by analyzing temporal correlations in hemodynamic signals occurring between different areas of the brain [79]. Perhaps counterintuitively, a number of rsfcMRI studies in the blind have found *decreased* functional connectivity between occipital cortex and nonvisual sensory processing areas of the brain [80–83]. For example, Yu and colleagues reported reduced functional connectivity between primary visual cortex and the rest of the brain [81] while Liu and colleagues [80] reported low rsfcMRI correlations between sensorimotor, auditory and multisensory cortices. Two more recent studies have used rsfcMRI analysis to provide further evidence regarding the nature of the reorganization of brain of blind individuals. Burton and colleagues found that (compared to normally sighted controls) early blind individuals exhibited decreased functional connectivity between auditory or somatosensory cortices and the visual cortex [84]. By contrast, increased functional connectivity was found between the occipital visual cortex and frontal as well as parietal regions of the brain. A similar result was also reported by Deen and coworkers, once again demonstrating comparatively enhanced functional connectivity between occipital cortical areas and frontal cortical areas implicated with executive control, with particular emphasis on working memory tasks [85].

The more recent findings of increased occipital cortical connectivity with higher order cortical areas may also help elucidate the nature of neuroplastic changes and behavioral adaptations with regards to not only non-visual sensory processing (i.e., hearing and touch), but also high level cognitive tasks such as memory and language [84,85]. These results raise interesting questions regarding the nature of brain reorganization resulting from early onset and longstanding visual deprivation and its relation to the compensatory behaviors observed in the blind. However, reconciling previous evidence of altered interactions within networks responsible for the processing of non-visual sensory information will require further study.

### The case of cortical/cerebral visual impairment (CVI)

It is important to recognize that the vast majority of scientific research investigating structural and functional brain changes resulting from profound visual deprivation has been carried out within the context of ocular causes of blindness. However, current evidence suggests that the leading etiology of pediatric visual impairment in developed countries is not due to ocular disease, but is rather caused by damage to the brain itself. This condition is known as cortical/cerebral visual impairment (CVI) [86]. In CVI, visual impairment results from pre- or perinatal damage to key cerebral structures implicated with the processing of visual information, as opposed to the eye itself [87]. Yet, despite the high prevalence of CVI and its potentially detrimental consequences on visual function and development, comparatively little research has been conducted on the developmental repercussions and neuroplastic compensatory mechanisms in these individuals.

Having previously described evidence outlining anatomical and functional changes uncovered in the case of ocular blindness, one can then ask by extension: what happens in the case when visual impairment or blindness is related to damage to the visual brain rather than the eyes? Furthermore, what is the fate of visual cortical areas that are damaged early on in development, and how do compensatory mechanisms develop within the context of CVI? It should be noted that there is great variability in terms of the visual impairment in individuals with CVI, varying from normal to profoundly impaired visual acuity, visual field defects, and impairments in visual cognitive processing such as motion perception, spatial localization, and way-finding [88,89]. In light of these deficits, it has been proposed that CVI may represent primarily a dysfunction of the spatial or “dorsal” visual processing stream [86]. The dorsal visual processing stream connects occipital visual areas with the parietal cortex and is responsible for spatial processing abilities. This is opposed to the ventral processing stream connecting occipital visual areas to the temporal cortex and typically associated with object recognition [90].

In a preliminary attempt to characterize structural brain changes occurring in CVI, Serdaroglu, Tekgul, Kitis, Serdaroglu, & Gökben (2004) reported that the degree of gross cerebral morphological changes detected by standard MRI techniques (such as grossly enlarged ventricles; referred to as periventricular leukomalacia or PVL) was correlated with the severity of this condition [91]. Indeed, the notion of an association between structural changes observed and the type of functional visual impairments observed in CVI has been proposed previously [92]. However, it is important to note that standard clinical MRI studies do not illustrate the full extent, nor the detailed architectural changes, that underlie the structural abnormalities associated with CVI. Furthermore, given the wide range of visual impairments observed in individuals with CVI, it is unclear how these deficits relate to underlying structural changes throughout the entire brain, particularly in terms of the division of labor between the dorsal and ventral visual processing streams.

**Figure 2:**
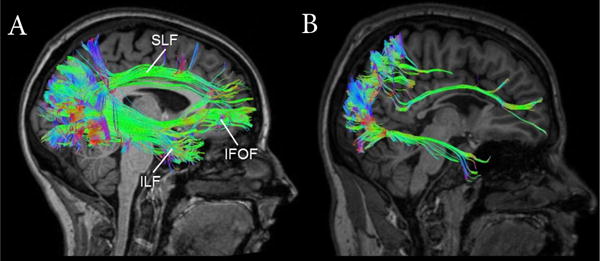
Diffusion based MRI reveals differences in white matter connectivity White matter tractography reconstruction in a normally-sighted control subject (**A**) compared to an age matched individual with CVI (**B**). In the control subject, the inferior longitudinal fasciculus (ILF), superior longitudinal fasciculus (SLF) and inferior fronto-occipital fasciculus (IFOF) are all evident. In contrast, marked reductions in each of these fasciculi is evident. Color scheme represents the direction of the white matter fibers (see Figure 1B).

Advanced neuroimaging techniques enable us to examine brain structure and anatomical pathways in a more detailed manner in the hopes of furthering our understanding of the neuroanatomical basis of CVI. Recent investigations reported differences in the neuroanatomical structure of individuals with CVI compared to individuals with normally developed brains. In particular, diffusion based MRI studies have revealed reductions in the integrity of specific white matter fibers responsible for the processing of information from primary visual areas to higher order cortical visual areas, such as the temporal, frontal, and parietal lobes (Figure 2). For example, the integrity (as measured by fractional anisotropy) of the neuroanatomical correlate of the ventral visual processing stream (the inferior longitudinal fasciculus or ILF) is significantly decreased in CVI compared to normally developed controls [93]. These observed alterations to the ILF have also been supported by a recent report indicating a severe reduction in the number of white matter fibers reconstructed in a variant of DTI in individuals with CVI [94].

Deficits in motion processing and spatial tasks are thought to be related to the dorsal stream, which is associated with the superior longitudinal fasciculus (SLF), while deficits in visual attention and eye movements are governed by frontal-occipital connections, namely the inferior fronto-occipital fasciculus (IFOF). Given that individuals with CVI have deficits in both types of visual processing, it can be hypothesized that the SLF and IFOF may be altered in CVI. Indeed, individuals with CVI show drastic reductions in the density and number of fibers of both the SLF and IFOF. Interestingly, in this same study, these reductions were not observed in ocular blind subjects [94,95].

Given the role supported by the ILF, SLF and IFOF in various aspects of visual processing (respectively, object, spatial, as well as visually guided attention and eye movement control), it seems plausible that the visual impairments observed in CVI can be associated (and even correlated, see [96]) with specific structural changes in white matter connectivity at the individual level. Indeed, these early findings using diffusion based MRI appear to support the notion that the brains of individuals with CVI show dramatic differences compared to normally developed brains as well as the case of ocular blind individuals.

While investigations using advanced neuroimaging in CVI are still preliminary, these early observations raise important questions not only with regard to the significance of associated links between neuroanatomical and clinical findings, but also how impairments in brain connectivity may translate to the education and rehabilitation of individuals living with blindness and profound visual impairment. For example, if indeed fundamental differences exist in how the brains of CVI and ocular blind children are connected, it would follow that perhaps the educational and rehabilitative strategies employed for one group may not be ideal for individuals in the other, and vice versa [97]. Thus, the simple diagnosis of visual impairment/blindness alone does not suffice in terms of determining an appropriate educational and/or rehabilitation strategy for an individual. Certainly, future work in this area appears warranted in order to better understand how underlying brain structure relates to optimal education and rehabilitative strategies. In the same way that detailed neuroimaging studies have helped us uncover the structural and functional changes that occur in the brain resulting from ocular blindness, and further, their relation to compensatory behaviors, it is hoped that similar structural and functional imaging studies will lead to important insights in children and adolescents with CVI. This may help to better understand the interrelationship between specific developmental deficits, underlying brain anatomy and function, as well as compensatory behavioral adaptations, and eventually allow for CVI-specific educational and rehabilitation strategies to be developed and tested more empirically.

## Conclusion

It is clear that individuals living with profound visual impairment have to make remarkable adjustments in order to remain functionally independent and integrated in a world that relies heavily on vision. In this review, we discuss the importance of neuroimaging technology and how it has helped uncover structural and functional changes within the brain. Further, we discuss how these changes relate to compensatory behaviors observed in individuals living with blindness. Structural neuroimaging modalities (particularly MRI-based approaches) have been instrumental in characterizing changes in brain regions implicated in the processing of non-visual sensory information such as hearing, touch, smell, and memory. These structural changes appear related to use dependency plasticity. In turn, functional neuroimaging has provided key evidence uncovering the role played by brain areas normally associated with the processing of visual information. Within the setting of blindness, the occipital cortex is recruited for the processing of nonvisual sensory information including touch, hearing, touch, smell, and also memory. Crucial is the fact that this recruitment appears to be intimately related to the compensatory abilities observed in early ocular blind individuals. In the case of developmental damage to the occipital cortex (such as in the case of CVI), the underlying neuroplastic changes and how they relate to compensatory behaviors remains less clear and await further investigation. In the end, understanding the conditions that promote neuroplastic changes within the brain, both in the setting of ocular and cortical/cerebral blindness, will be crucial to help inform (and potentially even individually tailor) educational and rehabilitation strategies for these individuals.

### Search strategy

A systematic search was conducted using the PubMed database (http://www.ncbi.nlm.nih.gov/pubmed) with the following keywords (MeSH Terms and All Fields): plasticity, blindness, visual deprivation. The search produced 4,892 articles limited to journals published in the English language. Adding the terms brain imaging and scanning resulted in 346 articles that were used for the purposes of this review. A secondary search was also conducted using the terms: electroencephalography (EEG), evoked related potential (ERP), magnetic resonance imaging (MRI), functional magnetic resonance imaging (fMRI), positron emission tomography (PET), diffusion based imaging, computed tomography (CT). These latter search results were limited to reviews and publications within the last 10 years.

## Figures and Tables

**Table 1:** Overview of structural and functional neuroimaging techniques.

Technique	Information Obtained	Advantages	Drawbacks
***Structural***			
Computed Tomography (CT)	Visualizationof gross brain abnormalities and bone structure	- Useful for imaging gross brain structure, malformations, as well as cerebral vascular accidents (e.g., hemorrhage) - Widely available, rapid image acquisition and processing	- Requires exposure to X-Ray radiation - Comparatively lower spatial resolution than MRI
Magnetic Resonance Imaging (MRI)	Visualization of brain soft tissue such as grey and white matter	- Good spatial resolution - Noninvasive despite exposure to high-intensity magnetic fields - Relatively accessible	- Cannot be used in subjects who have metallic implants (e.g., pacemakers) - Loud environment - Difficult for patients uncomfortable in enclosed spaces.
Diffusion-based MRI (e.g., Diffusion Tensor Imaging; DTI)	Detailed information regarding brain integrity, microstructure, and white matter connections	- Can quantify brain structural integrity as well as delineating white matter pathways connecting different regions of the brain.	- Complex image analyses required - Relatively long acquisition times - Sensitive to patient movement - Loud environment
***Functional***			
Functional MRI (fMRI)	Localization of brain activity associated with performing a cognitive task and/or behavior	- Good spatial resolution - Considered noninvasive despite exposure to exposure to high-intensity magnetic fields	- Limited temporal resolution ability - Loud environment - Indirect measure of brain activity
Positron Emission Tomography (PET)	Localization of brain activity as well as metabolism associated with performing a cognitive task and/or behavior	- Silent - Can track various metabolites in the brain such as glucose	- Comparatively poor spatial and temporal resolution compared to other techniques - Invasive due to required use of radioactive tracers - High costs and technical complexity - Limited to short tasks and radiation exposure limits repeated scans on same subject - Indirect measure of brain activity
Electroencephalogram (EEG) & Evoked Related Potentials (ERP)	Direct recording of underlying electrical brain activity associated with a cognitive task and/or behavior	- Good temporal resolution - Silent - Relatively tolerant of subject movement - Noninvasive - Low cost of use - Brain activity can be directly associated to a stimulus or event (e.g., triggered visual stimulus)	- Poor spatial resolution compared to fMRI - Analysis of acquired data can be very complex
